# Antimicrobial Resistant Isolates in Surgical and Bite Wounds in Dogs and Cats: A 12-Year Retrospective Analysis

**DOI:** 10.3390/ani16030501

**Published:** 2026-02-05

**Authors:** Davide Danieli, Michela Amadori, Sara Crimi, Federica Pregnolato, Chiara Caruso, Graziana Gambino, Giovanni Re, Cristina Vercelli

**Affiliations:** 1 Department of Veterinary Sciences, University of Turin, 10095 Grugliasco, Italy; davide.danieli@unito.it (D.D.); sara.crimi@unito.it (S.C.); graziana.gambino@unito.it (G.G.); giovanni.re@unito.it (G.R.); cristina.vercelli@unito.it (C.V.); 2 Department of Public Health and Pediatric Sciences, University of Turin, 10126 Turin, Italy; 3 Centro Veterinario Torinese (CVT) Hospital, 10098 Rivoli, Italy; federica.pregnolato@cvtrivoli.it (F.P.); caruso@cvtleclinicheveterinarie.it (C.C.)

**Keywords:** antimicrobial resistance, companion animals, surgical site infections, bite wounds

## Abstract

Antimicrobial resistance (AMR) is an increasingly concern in both human and veterinary medicine, complicating the management of common clinical infections. Surgical site infections (SSIs) and bite wounds in pets are especially challenging due to their polymicrobial nature and multidrug-resistant strains. Laboratory records from a referral veterinary hospital in northwestern Italy were retrospectively analyzed. Bacterial cultures and antimicrobial susceptibility testing results from canine and feline surgical and bite wounds collected between 2013 and 2024 were reviewed. Data were analyzed descriptively to identify bacterial distribution and resistance trends. A total of 35 isolates (23 dogs; 12 cats) were obtained from 26 surgical and 9 bite wounds. In dogs, *Enterobacter cloacae*, *Escherichia coli*, *Klebsiella pneumoniae*, and *Staphylococcus pseudintermedius* were most frequently identified. In cats, *Enterobacter cloacae*, *Escherichia coli*, and *Pasteurella multocida* predominated. High resistance rates were recorded for amoxicillin–clavulanate, cephalexin, enrofloxacin, and marbofloxacin. Methicillin resistance emerged in most *Staphylococcus aureus* and several *S. pseudintermedius* isolates, while subsets of *E. coli* and *K. pneumoniae* were ESBL. Resistance to carbapenems remained low. Although samples were limited, these findings underscore the ongoing impact of AMR in surgical and bite wounds and highlight the importance of implementing rigorous antimicrobial stewardship practices in veterinary medicine.

## Simple Summary

Antimicrobial resistance is one of the major health challenges of this century, affecting both animals and humans and making it a true One Health issue. Using antibiotics carefully and only when needed is essential to slow the spread of resistant bacteria and to preserve the effectiveness of these drugs. This study investigated antibiotic resistance in dogs and cats with infected surgical wounds or bite wounds—two of the most common reasons pets receive antibiotics—in a veterinary hospital located in northeastern Italy. The findings reveal a concerning situation: many of the antibiotics most frequently prescribed in small-animal practice showed high resistance rates, and a large proportion of treatments were empirical, meaning they were not guided by laboratory testing of bacterial susceptibility. Despite this, the data also highlighted an encouraging point: resistance to “last-resort” antibiotics remained low, suggesting that these critically important drugs are still effective when needed. Antibiotic resistance is now a widespread and growing problem worldwide. Responsible, well-targeted, and protocol-driven antimicrobial use is essential to limit its expansion, reduce the selective pressure that favors resistant bacteria, and protect the therapeutic value of antibiotics for both animals and humans.

## 1. Introduction

Antimicrobial resistance (AMR) is today one of the principal global health emergencies, with profound impacts on human, animal, and environmental health, fully consistent with the One Health concept [1]. AMR is defined as the ability of bacteria to survive exposure to an antibiotic, leading to partial or complete treatment failure. This loss of efficacy contributes to higher incidence and mortality in human and veterinary infections [1,2,3]. Despite numerous stewardship efforts, antibiotic consumption remains high in both medicines, thus fueling a worrying trend: the latest estimates suggest that, in the absence of effective interventions, AMR could claim up to 10 million lives annually by 2050 [4].

Several international bodies have implemented AMR-control measures, including the World Organization for Animal Health (WOAH, ex OIE) and the European Union, which enforces strict prescription and surveillance rules through agencies such as the European Medicines Agency (EMA), the European Center for Disease Prevention and Control (ECDC), the European Food Safety Authority (EFSA), and the European Surveillance of Veterinary Antimicrobial Consumption (ESVAC) [1,5]. In 2013, the EMA established the first expert group in this area—the Antimicrobial Advice ad hoc Expert Group (AMEG)—which created a four-category system to assess the risk of resistance transfer from animals to humans [6].

The World Health Organization (WHO) similarly prioritizes antimicrobial classes through its definition of Critically Important Antimicrobials (CIAs), defined as classes essential for severe human infections and implicated in resistance exchange with non-human sources. This framework guides prudent-use policies across sectors [5]. In 2024, the CIA framework evolved into the Medically Important Antimicrobials List (MIA), a more comprehensive risk-management tool that integrates clinical relevance with the risk of resistance emerging from non-human (animal or environmental) use and subsequently affecting human health [7].

In companion animal practice, the role of pets in AMR transmission is increasingly recognized. Close cohabitation between animals and owners, coupled with direct contact through bites, scratches, or licking, facilitates the exchange of resistant bacteria. Indiscriminate or inappropriate antimicrobial use in small animals has enhanced selection of multidrug-resistant (MDR) strains, reducing susceptibility across entire drug classes [3].

Effective AMR control requires optimal management of bacterial infections. However, standardized protocols for prevention, diagnosis, and treatment are still limited in the field of companion animal medicine, thereby underscoring the importance of antimicrobial stewardship programs (ASPs). These programs promote targeted antibiotic use that balances patient needs with public health goals [3,8]. The obstacles to these practices include regulatory barriers, lack of training, owner expectations, and financial constraints linked to microbiological diagnostics and susceptibility testing [1,9].

Surgical-site infections (SSIs) constitute the most common nosocomial infection in small animals, with incidence ranging from 0.8% to 18.1% [10,11,12,13]. Frequently sustained by MDR bacteria, SSIs increase morbidity, mortality, hospitalization period, and management costs, with costs rising by roughly 142% in complicated cases [14,15].

It has been reported that common pathogens include opportunistic skin and environmental bacteria such as *Staphylococcus* spp., *Pseudomonas* spp., *Klebsiella* spp., *Citrobacter* spp., *Enterobacter* spp., and *Enterococcus* spp. [16].

Risk factors for SSI development encompass pathogen-related attributes (such as load, virulence, and synergism), environmental variables (such as operating-room asepsis and number of personnel), patient-specific factors (i.e., age, immune status, comorbidities, hypothermia, and hyperglycaemia), and surgical-technical aspects (for example, procedure duration, implants, dead space, and suture tension) [10,11,13,14,17,18].

Pre-operative prophylaxis can reduce SSIs risk [19], whereas post-operative antibiotic use in clean surgery is widely debated. Major guidelines discourage routine administration of antibiotic drugs for prophylaxis in the absence of predisposing conditions, as it does not reduce infection rates and contributes to unnecessary antibiotic use [12,14]. Similarly, Regulation (EU) 2019/6 on veterinary medicinal products establishes strict rules on the prophylactic and metaphylactic use of antimicrobials, clearly limiting their administration and reinforcing the principle that antibiotics should not be used routinely in the absence of specific clinical indications [20].

Bite wounds likewise represent a frequent source of infection, accounting for 10–15% of veterinary emergencies. Regardless of their clinical appearance, these lesions are considered contaminated by the attacker’s oral microbiota, the victim’s skin microbiota, and the environment [21,22,23]. Davis and Weese (2022) analyzed the oral microbiota of dogs and cats using gene sequencing technology [24]. Considering the oral polymicrobial community, a higher prevalence of Gram-negative bacteria was present in healthy pets than the microbiota shifted to Gram-positive bacteria (e.g., Firmicutes in dogs) in those companion animals suffering from inflammation, such as periodontitis [24]. Gram-negative bacteria such as *Neisseria* and *Bergeyella* prevailed in clinically healthy dogs, while *Pasteurella*, *Thermomonas*, and *Moraxella* prevailed in healthy cats [24]. Conversely, the oral microbiota in healthy people has a prevalence of Gram-positive bacteria (e.g., *Streptococcus* spp.), while in cases of periodontitis, Gram-negative bacteria such as *Treponema denticola*, *Porphyromonas gingivalis*, and *Tannerella forsythia* prevail. People share 16.4% of the oral taxa of dogs [24]. When evaluating the best antibiotic treatment to administer following a bite wound, the microorganisms influence the antibiotic therapy, but several other factors must also be taken into account: canine bites tend to be more extensive due to crushing and laceration, whereas feline puncture wounds can inoculate bacteria deeply [24].

Complications include dead-space formation and, in severe cases, progression to systemic inflammatory response syndrome or sepsis. Isolated pathogens are various, like *Pasteurella* spp., *Neisseria* spp., *Staphylococcus pseudintermedius*, *Escherichia coli*, *Klebsiella* spp., *Pseudomonas* spp., *Enterococcus* spp., *Streptococcus* spp., *Chryseobacterium* spp., *Moraxella* spp., *Campylobacter* spp., and *Corynebacterium* spp. [25,26], but variations in oral microbiota composition can occur according to environmental, dietary factors, and oral inflammation [24,26].

Pharmacological treatment supports but cannot replace meticulous surgical management, including debridement, lavage, and choice of primary versus secondary closure [24]. Antibiotic therapy should be selective: these drugs should be reserved only for patients with a high risk of infection, like animals with deep wounds, extensive tissue laceration, or immunocompromised status. In fact, every time an antibiotic is administered to a pet, bacteria can develop antibiotic resistance mechanisms that can spread among pet owners, other animals, and the environment [3]. Nevertheless, empirical antimicrobial use remains common, occurring in 86–88% of cases, often without bacterial isolation and susceptibility testing, and it seems that it is mainly due to economic constraints [23,27].

Surgical infections and bite wounds thus pose major clinical challenges amid rising AMR, and demand rational antimicrobial therapy. The pervasive use of empirical antibiotics combined with the dissemination of resistant strains—including methicillin-resistant *Staphylococcus aureus* (MRSA), *Staphylococcus pseudintermedius* (MRSP), and extended-spectrum beta-lactamase (ESBL)-producing *E. coli*—has eroded first-line treatment options [28,29].

Based on these considerations, the present study aimed to retrospectively describe the twelve-year (2013–2024) AMR trends in bacteria isolated from surgical-site and bite-wound infections in dogs and cats in a referral veterinary hospital in Northwestern Italy. The aim was to identify prevalent pathogens and resistance patterns to support targeted therapy and inform future ASP strategies.

## 2. Materials and Methods

### 2.1. Animals

The medical records of canine and feline patients referred to a veterinary teaching hospital in Northwestern Italy between 1 January 2013 and 31 December 2024 were retrospectively reviewed. Animals were eligible for inclusion if they met all the following criteria:

(A) species: dog or cat;
(B) clinical diagnosis of either a bite wound (BW) or a surgical site infection (SSI) during the study period;
(C) availability of bacterial culture and antimicrobial susceptibility testing performed on samples collected directly from the wound site.

Bite wounds were defined as traumatic skin and/or soft tissue lesions inflicted by dogs or cats and documented as such in the clinical records based on patient history, physical examination findings, and lesion characteristics. Wounds of unknown origin or resulting from other traumatic events (e.g., vehicular trauma, foreign bodies, or environmental injuries) were excluded.

Surgical site infections were included when a previous surgical procedure was documented in the medical record and at least one clinical sign consistent with infection was reported. These signs included erythema, edema, pain, local heat, purulent or serous exudate, wound dehiscence, and/or delayed wound healing. SSIs were identified based on the attending clinician’s clinical judgment, in accordance with routine clinical practice at the time of case presentation. Given the retrospective nature of the study, no additional standardized or time-based diagnostic criteria for SSI were retrospectively applied.

Cases were excluded if microbiological culture and antimicrobial susceptibility testing were not performed, if samples were not collected from the wound site, or if clinical or laboratory records were incomplete. When multiple samples or repeated cultures were available from the same patient and infectious episode, only data related to the same clinical event were considered.

For each included patient, the following data were extracted from the medical records: species, age, sex, reproductive status, clinical history, type of wound (BW or SSI), and information on current or prior pharmacological treatments, when available. Owing to the retrospective design of the study, diagnostic approaches, treatment protocols, and case management were not standardized.

Written informed consent for diagnostic and therapeutic procedures was obtained from all owners at the time of clinical admission, in accordance with institutional policies.

#### 2.1.1. Sex

Animals were divided into males and females, identifying those that were intact and those that had been sterilized.

#### 2.1.2. Age

Animals were stratified as young (0–2 years), adult (2–8 years), or senior (>8 years).

### 2.2. Samples

Wound swabs were collected from infected lesions—classified as SSIs or bite wounds—during routine clinical activity between 2013 and 2024. After gentle cleansing with sterile saline (no antiseptics to avoid growth inhibition), sterile swabs were applied directly onto or into the lesion and placed in appropriate transport medium at room temperature. Samples were shipped to an external (quality-certified) diagnostic laboratory and processed within 24 h for bacterial growth. The samples were cultured on media under aerobic, microaerophilic, and anaerobic conditions for 2–6 days, depending on the rate of bacterial growth. If there was no bacterial growth within this period, the sample was considered negative. In the event of bacterial proliferation, the microorganisms were subjected to identification and automated broth microdilution for antimicrobial susceptibility testing (antibiogram) according to standard veterinary microbiology protocols.

Pathogen identification was performed using VITEK-MS mass spectrometry (bioMérieux, Craponne, France), based on matrix-assisted laser desorption/ionization time-of-flight (MALDI-TOF) technology, according to the manufacturer’s protocol. Specifically, the sample is prepared by mixing a bacterial colony with a special matrix. The sample is then ionized using a laser beam. The absorption spectrum of the individual ionized fragments depends on the speed at which they reach the analysis membrane, in relation to their molecular size. An antibiogram was performed with VITEK 2 COMPACT. The bacterial colonies are suspended in 0.9% NaCl solution at a standardized concentration. Then, a specific card is inoculated with the bacterial suspension and placed in an incubator/reader. The instrument then measures bacterial growth for 18 h, comparing it with standard curves. The susceptibility testing was interpreted according to CLSI guidelines specific to companion animals and EUCAST guidelines for reference data not available in veterinary medicine. All isolates were evaluated for MDR. *Enterobacterales* were screened for extended-spectrum β-lactamase (ESBL) production, while staphylococci were assessed for methicillin resistance by oxacillin/cefoxitin screening. All diagnostic procedures were performed exclusively for clinical decision-making in privately owned animals, with costs borne entirely by the owners. Given the retrospective nature of the study and the absence of a predefined research protocol, microbiological investigations were limited to pathogen identification and phenotypic antimicrobial susceptibility testing routinely required for clinical management. Therefore, no molecular or genetic analyses aimed at characterizing antimicrobial resistance determinants were performed.

### 2.3. Data Analysis

Antibiogram data were entered into an Excel file (Microsoft Corporation, Redmond, WA, USA), recording species, breed, sex, age, microbiology report date, isolated pathogens, susceptibility profiles, and minimum inhibitory concentrations (MICs—µg/mL) when available. Descriptive statistics were performed using pivot tables to group data, calculate percentages, and generate graphs.

Repeat swabs from the same patient within 2 months were included only if susceptibility profiles differed, to avoid duplicate data.

## 3. Results

Between 2013 and 2024, 41 clinical cases met the inclusion criteria. Positive cultures were obtained from 29 patients (70.7%), while 12 produced negative swabs (29.3%). Of the 29 positive swabs, 18 (62.1%) were from dogs and 11 (37.9%) from cats. Among dogs, 14 isolates (77.8% of positive dogs; 48.3% overall) were associated with SSIs and 4 (22.2%; 13.8% overall) with bite wounds. Among cats, 7 swabs (63.6% of positive cats; 24.1% overall) related to SSIs and 4 (36.4%; 13.8% overall) to bite wounds.

### 3.1. Sex Distribution

**Figure 1:**
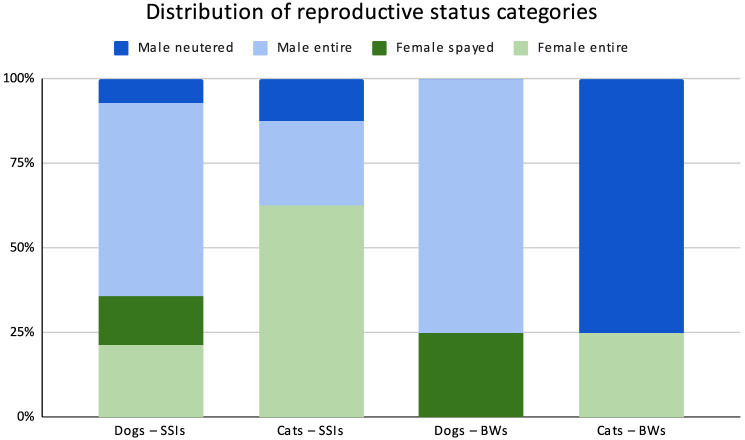
Stacked bar chart showing the percentage distribution (100% per bar) of reproductive status categories (female entire, female spayed, male entire, and male neutered) in dogs and cats affected by surgical site infections (SSI) and bite wounds (BW). Each bar represents a single species–condition group (dogs–SSI, cats–SSI, dogs–BW, and cats–BW) and is normalized to 100%, allowing comparison of relative proportions independently of group size. Absolute case numbers for each category are reported in Table 1.

The distribution of reproductive status among animals with surgical site infections (SSI) and infected bite wounds (BW) is shown in Figure 1 and Table 1.

Of 21 SSI patients, there were 14 dogs—5 females (3 entire, 2 spayed) and 9 males (8 entire, 1 neutered)—and 7 cats—5 intact females and 3 males (2 entire, 1 neutered). Among infected-bite patients, there were 4 dogs—1 spayed female and 3 intact males—and 4 cats—1 intact female, 3 neutered males.

### 3.2. Age Distribution

**Figure 2:**
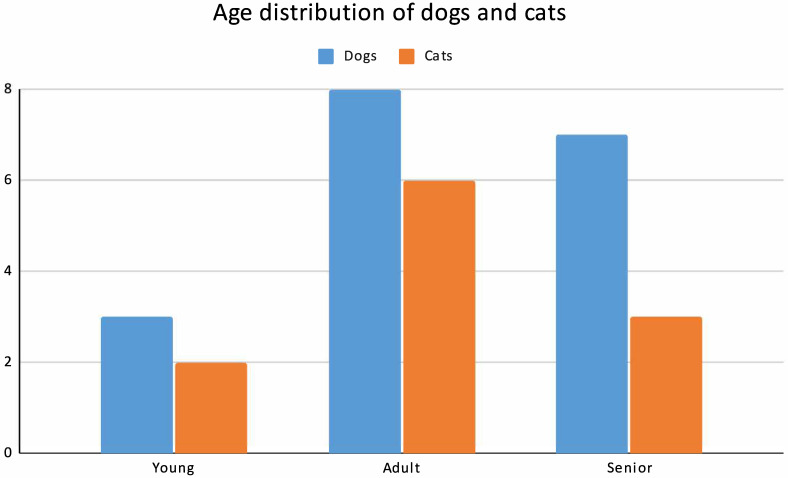
Grouped bar chart displaying the number of dogs and cats classified as young, adult, or senior. Each bar represents the absolute count of animals per species within each age category: animals were stratified as young (0–2 years), adult (2–8 years), or senior (>8 years).

The age distribution of the study population is shown in Figure 2. Among dogs (*n* = 18), 3 were classified as young, 8 as adult, and 7 as senior, whereas among cats (*n* = 11), 2 were young, 6 adult, and 3 senior.

### 3.3. Isolated Microorganisms

Fifteen different bacteria were isolated (35 total isolates, exceeding patient number due to four double and one triple infection): 23 canine and 12 feline. In decreasing order of frequency: *Escherichia coli* (20.0%), *Klebsiella pneumoniae* (14.2%), *Enterobacter cloacae* (14.2%), *Staphylococcus intermedius/pseudintermedius* (8.5%), *Pasteurella multocida* (5.7%), *Enterococcus faecalis* (5.7%), *Proteus mirabilis* (5.7%), *Serratia marcescens* (5.7%), *Klebsiella oxytoca* (2.8%), *Clostridium tertium* (2.8%), *Salmonella enterica* subsp. *enterica* (2.8%), *Streptococcus canis* (2.8%), *Staphylococcus aureus* (2.8%), *Burkholderia contaminans* (2.8%), and *Stephanoascus ciferrii* (2.8%).

**Figure 3:**
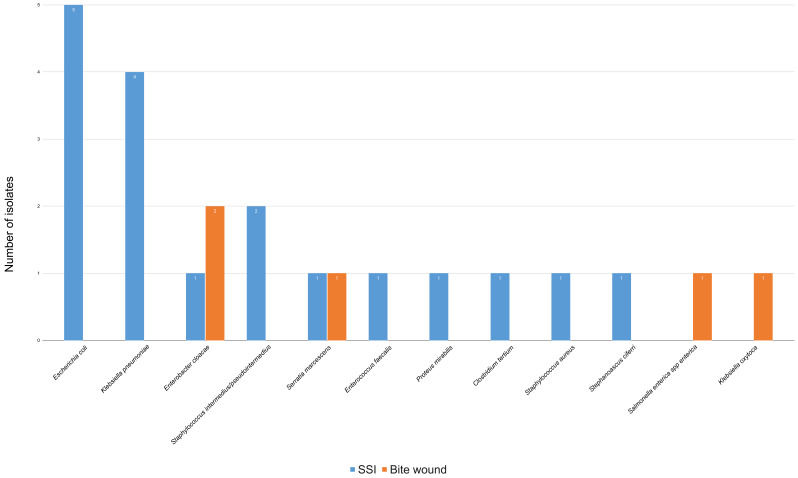
Distribution of bacterial isolates from canine patients, categorized by wound type: bite wounds (*n* = 5) and SSIs (*n* = 18). Each column represents a specific bacterium, and the color represents SSIs or bite wounds. The number of bacterial isolates differs from the number of patients, as multiple isolates were obtained from some cases.

**Figure 4:**
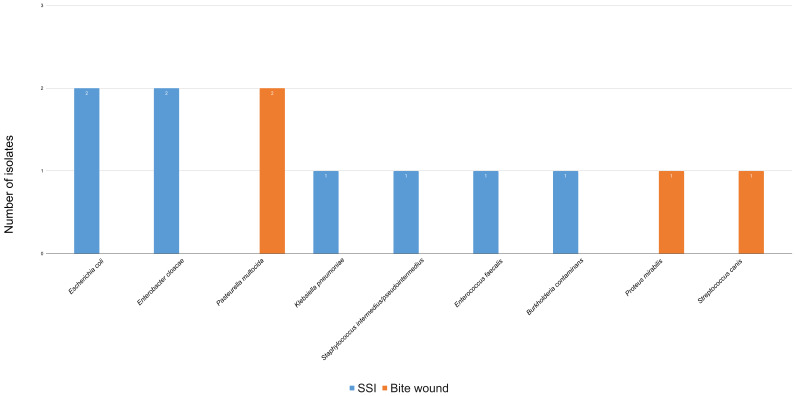
Distribution of bacterial isolates from feline patients, categorized by wound type: bite wounds (*n* = 4) and surgical site infections (SSIs) (*n* = 8). Each column represents a specific bacterium, and the color represents SSIs or bite wounds. The number of bacterial isolates exceeds the number of patients, as multiple isolates were obtained from some cases.

Multiple infections occurred in four canine cases (1 *Klebsiella oxytoca* and *Salmonella Enterica spp Enterica*; 1 *Staphylococcus intermedius/pseudointermedius* and *E. coli*; 1 *Klebsiella pneumoniae* and *Clostridium tertium;* 1 *Klebsiella pneumoniae*, *Staphylococcus intermedius/pseudointermedius,* and *Stephanoascus ciferrii*) and one feline case (*1 E. coli* and *Enterococcus faecalis*). The distribution of bacterial isolates by wound type in canine and feline patients is shown in Figure 3 and Figure 4, respectively.

In dogs, the three most frequent isolates were *E. coli* (5/23; 21.7%), *K. pneumoniae* (4/23; 17.4%), and *E. cloacae* (3/23; 13.0%). In cats: *E. coli*, *E. cloacae*, and *P. multocida* (each 2/12; 16.7%). Only bacterial species with the highest prevalence were considered for further discussion; single-occurrence isolates were no longer cited in the manuscript.

### 3.4. Resistance in Canine Isolates

**Figure 5:**
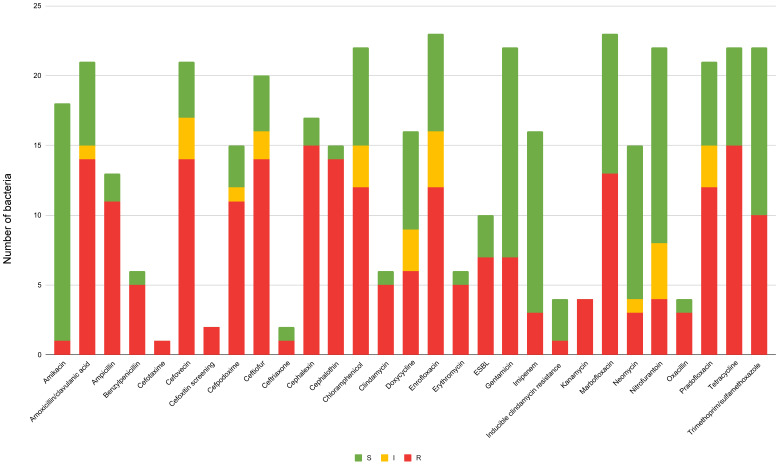
Antibiotic susceptibility profiles of bacterial isolates from canine samples. Each column represents a single antimicrobial agent, with stacked bars showing the proportion of isolates classified as susceptible (S, green), intermediate (I, yellow), or resistant (R, red). ESBL = extended-spectrum beta-lactamase-producing isolates. The graph provides a visual summary of resistance patterns across all tested antibiotics. Susceptibility was determined based on MIC-derived breakpoints. The susceptibility testing was interpreted according to CLSI guidelines specific to companion animals and EUCAST guidelines for reference data not available in veterinary medicine.

Antimicrobial susceptibility profiles of the three most frequently isolated bacterial species from canine samples are summarized in Table 2 and a visual overview of resistance patterns across all tested antimicrobial agents is provided in Figure 5. The phenomenon of AMR was particularly high for β-lactams, penicillins, and first-generation cephalosporins. *Escherichia coli* showed 80% (4/5) resistance to ampicillin, cefalotin, chloramphenicol, enrofloxacin, marbofloxacin, and pradofloxacin; 60% (3/5) to amoxicillin-clavulanate, cephalexin, cefovecin, cefpodoxime, ceftiofur, doxycycline, tetracycline, and trimethoprim-sulfamethoxazole. In contrast, all *E. coli* remained susceptible to amikacin and nitrofurantoin, and high susceptibility rates were also observed for gentamicin and neomycin (4/5; 80%). Most importantly, all isolated *E. coli* have been identified as sensitive to imipenem (5/5; 100%). *K. pneumoniae* was fully resistant to ampicillin and cephalexin; 75% (3/4) resistant to cefovecin, cefpodoxime, ceftiofur, enrofloxacin, marbofloxacin, and tetracycline, yet fully susceptible to amikacin, and partially susceptible to gentamicin (3/4; 75%). In this case, total sensitivity to imipenem was demonstrated. *E. cloacae* displayed complete resistance (3/3; 100%) to cephalexin, cefalotin, and ceftiofur, while remaining 100% (3/3) susceptible to amikacin, amoxicillin-clavulanate, gentamicin, neomycin, and tetracycline. Among the three bacterial strains examined, only *E. coli* and *K. pneumoniae* were tested for ESBL production. ESBL positivity was detected in 3 out of 5 *E. coli* isolates and in 3 out of 4 *K. pneumoniae* isolates.

### 3.5. Resistance in Feline Isolates

**Figure 6:**
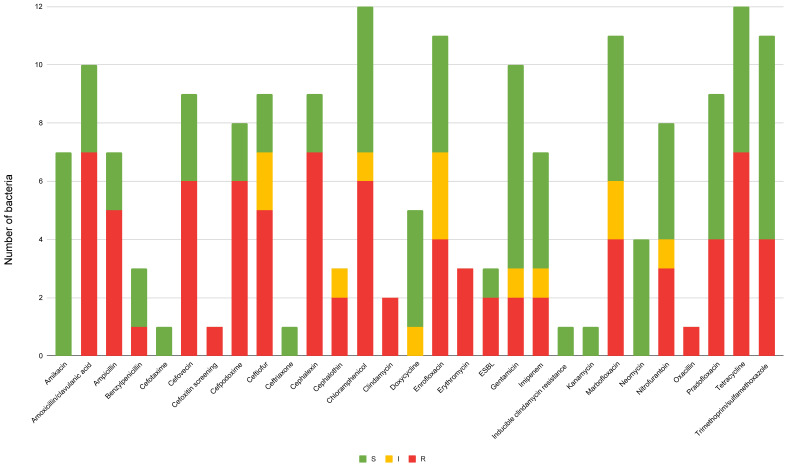
Antibiotic susceptibility profiles of bacterial isolates from feline samples. Each column represents a single antimicrobial agent, with stacked bars showing the proportion of isolates classified as susceptible (S, green), intermediate (I, yellow), or resistant (R, red). ESBL = extended-spectrum beta-lactamase-producing isolates. The graph provides a visual summary of resistance patterns across all tested antibiotics. Susceptibility was determined based on MIC-derived breakpoints. The susceptibility testing was interpreted according to CLSI guidelines specific to companion animals and EUCAST guidelines for reference data not available in veterinary medicine.

Antimicrobial susceptibility profiles of the three most frequently isolated bacterial species from canine samples are summarized in Table 3 and a visual overview of resistance patterns across all tested antimicrobial agents is provided in Figure 6. It is possible to appreciate a similar trend of resistance against β-lactams, similar to what was already described for canine samples. The number of samples analyzed for cats was limited, so the authors indicated the percentage of susceptibility to the antibiotic considered for each specific bacterium, emphasizing the number of specimens considered in each case. *Escherichia coli* isolates were resistant to ampicillin and cephalexin but fully susceptible to amikacin, doxycycline, gentamicin, neomycin, nitrofurantoin, and tetracycline.

Both *P. multocida* isolates exhibited pronounced susceptibility, including to amoxicillin-clavulanate, cephalexin, ceftiofur, chloramphenicol, enrofloxacin, marbofloxacin, neomycin, pradofloxacin, tetracycline, and trimethoprim-sulfamethoxazole.

Regarding *E. cloacae*, marked resistance to cephalosporins was observed, with both isolates resistant to cephalexin (2/2; 100%), cefpodoxime (2/2; 100%), and ceftiofur (2/2; 100%), as well as to amoxicillin-clavulanic acid (2/2; 100%), chloramphenicol (2/2; 100%), enrofloxacin (2/2; 100%), gentamicin (2/2; 100%), marbofloxacin (2/2; 100%), nitrofurantoin (2/2; 100%), and tetracycline (2/2; 100%). Only amikacin demonstrated high efficacy (2/2; 100%) against the isolated strains.

Overall, the summary histogram (Figure 6) shows a trend toward generalized resistance against β-lactams, with aminoglycosides, doxycycline, pradofloxacin, and trimethoprim-sulfamethoxazole maintaining good activity profiles. Imipenem was found to be sensitive in 4 out of 7 isolates (≈57%).

### 3.6. Antibiotic Treatment

All patients included in this study received antibiotic therapy, selected by the attending veterinarian based on the clinical presentation and the professional experience of the clinician in charge. All animals with surgical site infections (SSIs) or bite wounds also received local treatment with various products, including honey or a collagenase- and chloramphenicol-based ointment, at the discretion of the attending veterinarian.

Even the 12 animals that were subsequently found to be negative in antimicrobial susceptibility testing received empirical antibiotic therapy with amoxicillin–clavulanic acid or ampicillin, as determined by the treating veterinarian.

Dogs with SSIs (*n* = 14) received intravenous ampicillin in eight cases, oral marbofloxacin in two cases, and oral amoxicillin–clavulanic acid in four cases. Dogs with bite wounds (*n* = 4) received oral marbofloxacin in all four cases. Cats with SSIs (*n* = 7) received intravenous ampicillin in five cases and oral marbofloxacin in two cases, whereas cats with bite wounds (*n* = 4) received oral cefovecin in all four cases (Table 4).

## 4. Discussion

The present study aimed to describe the twelve-year trend (from the beginning of 2013 to the end of 2024) of AMR in bacteria isolated from SSIs and bite wounds in dogs and cats, referred to a veterinary hospital in Northwestern Italy. The goal was to collect specific data about the pathogens isolated in SSIs and bite wound patients, understanding the resistant profiles of isolated bacteria.

The focus of the present study was specifically on these topics, in fact, as these are among the most frequent causes of antibiotic use in veterinary patients, as recently reported in the literature [30]. This represented a change since it was reported that 10 years ago in Europe, the reasons to use antibiotics were dermatological, respiratory, urinary tract, and periodontal diseases, both in canine and feline patients, with slight variations in the percentage in the two species [31]. The most recent report highlighted that the indications for antibiotic use in dogs were mainly addressed to treat diarrhea (18.4%), wound infection/abscess/bite injury (16.0%), and dental treatment (8.2%), while in cats, wound infection/abscess/bite injury (23.3%) represents the most important reason to treat patients with antibiotics, followed by dental treatment (21.2%) and upper respiratory tract infections (16.7%) [30].

The findings of this study suggest an epidemiological trend of antimicrobial resistance (AMR), consistent with trends documented in both human and veterinary medicine. The detection of MDR strains (such as MRSA, MRSP, and ESBL-producing organisms) underscores the increasing challenge of empiric management of infections and highlights the necessity of microbiology-guided antimicrobial stewardship practices.

Indeed, the inter-species and cross-sector dissemination of MDR pathogens is well documented. For example, MRSA, MRSP, and ESBL-producing *Enterobacteriaceae* are increasingly isolated from companion animals, with evidence for shared genetic lineages between human and animal hosts [32,33,34]. In veterinary practice, the outbreaks of MRSA and MRSP, ESBL-producing *E. coli*, and MDR *Salmonella* have been described as illustrative of the zoonotic and One Health dimensions of AMR [33]. The spread of these bacteria can compromise clinical outcomes in pets, making it more difficult for them to heal and necessitating the use of second- or third-line antibiotics (AMEG C and B, respectively) that can more easily select for resistant strains [3]. In addition, sites of bacterial infections, such as SSIs and bite wounds, can facilitate the spread of AMR by pet owners through physical contact with their pets, and this risk is much higher in the case of bacteria with marked antibiotic resistance characteristics. In fact, due to the close interaction between pets and their owners, severe bacterial infections can develop in humans, compromising public health and also spreading the same bacteria and antibiotic-resistant genes into the shared environment [3].

Given this background, the presence of MDR isolates in our study setting reinforces the imperative for ASPs that are formed by a close cooperation among microbiologists, pharmacologists, and clinicians [2]. In both human and veterinary medicine, ASP interventions—such as appropriate antibiotic selection, dosing, route, duration, de-escalation, and infection prevention and control—have been advocated to preserve antimicrobial efficacy and mitigate resistance emergence [35]. Thus, the detection of MRSA, MRSP, and ESBL-producing strains in our study not only mirrors the international literature but also emphasizes that empiric therapy in such contexts is increasingly unreliable. Microbiology-guided prescribing—by using culture and susceptibility data, local antibiograms, and sequential review of therapy—is therefore critical to optimize therapeutic success and limit further AMR expansion [3,29,36].

It is evident how low the number of susceptibility tests performed is in such a long period of time, specifically considering the type of infection. In fact, this small animal hospital is a reference center for emergencies and elective clinical procedures. It can host an average of 2000 patients per year, and it is possible to identify that each year about 40 patients are referred to the emergency department for bite wounds and trauma (i.e., accidents, collisions, falls) that can cause serious injuries to the skin, resulting in infections. Considering these factors, a vast majority of patients have been treated in an empirical way using amoxicillin + clavulanic acid, ampicillin, marbofloxacin, or cefovecin, and in only 41 cases has the susceptibility testing been performed. This is important data in itself, thus highlighting that the use of such a diagnostic tool is normally underused.

Out of 41 clinical cases, 29 (70.7%) yielded positive cultures, with 62% of them originating from dogs and 38% from cats. SSIs accounted for 72.4% of the total isolates, and this confirms that susceptibility testing is a diagnostic tool used more when the situation immediately appears more complicated and worrying (in this situation, in patients who underwent a surgical procedure) and underlines their relevance among the most common nosocomial infections in companion animals.

A total of 15 different pathogens were identified (35 isolates in total, exceeding patient number due to four double and one triple infection), primarily represented by *Escherichia coli* (20%), *Klebsiella pneumoniae* (14%), and *Enterobacter cloacae* (14%). The predominance of *Enterobacterales* as major SSI pathogens aligns with European and North American studies [10,16], which reported rates ranging from 35% to 60% of SSI isolates. Despite the limited number of cases, a notable prevalence of Gram-negative opportunistic pathogens—such as *E. cloacae*, *E. coli*, *K. pneumoniae*, *Pasteurella multocida*, and *Proteus mirabilis*—was observed in both wound types. This finding is consistent with the literature [16,24] and supports the hypothesis of a mixed microbial origin (endogenous sources such as skin and mucosae, and exogenous sources such as the environment, surgical tools, or bite trauma).

The high frequency of isolates typically associated with bite wounds, including *P. multocida*, *S. intermedius/pseudintermedius*, and *Streptococcus canis*, confirms prior observations regarding the distinct nature of oral microbiota—predominantly composed of microaerophilic or facultative anaerobic bacteria [26].

In dogs, 80% of *E. coli* isolates were resistant to ampicillin, cephalothin, and fluoroquinolones, while showing full susceptibility to amikacin, imipenem, and nitrofurantoin (100%).

*Klebsiella pneumoniae* exhibited complete resistance to penicillin and first-generation cephalosporins but retained full susceptibility to amikacin. Three *E. coli* strains (60%) and one *K. pneumoniae* strain (25%) were ESBL producers; this is particularly concerning, as these enzymes confer extensive resistance to β-lactam antibiotics.

In cats, *E. coli* demonstrated a similar pattern (resistance to ampicillin and cephalexin), while retaining full susceptibility to aminoglycosides, doxycycline, and nitrofurantoin.

*P. multocida*, commonly part of feline oral microbiota, showed susceptibility to most tested drugs. Conversely, feline *E. cloacae* was highly resistant, with amikacin being the only consistently effective option.

In both species, an alarming rate of resistance to fluoroquinolones was noted, thus confirming the selective pressure associated with their use in veterinary medicine and highlighting the need to restrict their application, in accordance with the EMA classification (Category B: “Restrict”). Overall, in the present study, a trend of generalized resistance to β-lactam antibiotics is shown, whereas aminoglycosides (i.e., amikacin and gentamicin), doxycycline, pradofloxacin, and trimethoprim/sulfamethoxazole retained good activity. This resistance profile reflects the selective pressures seen in settings where empirical therapy and postoperative prophylaxis are still widely adopted [3,8,13].

The low prevalence of carbapenem resistance is encouraging, suggesting that these drugs (considered life-saving in human medicine) are not compromised in the veterinary field, indicating that companion animals do not affect their use in human medicine. In fact, imipenem and other carbapenems must not be used to treat veterinary patients, according to the AMEG classification, which places these antibiotics in category A (“Avoid”) among antibiotics prohibited in veterinary medicine precisely because they represent the last resort antibiotic treatments for human patients and are used in hospitals to treat serious bacterial infections. Nevertheless, these molecules are inserted in susceptibility testing as markers for carbapenemase-producing bacteria, and it is important to identify these bacteria, especially *E. coli* and *K. pneumoniae*, which are the most represented carbapenemase-producing *Enterobacterales* found in companion animals, alongside *Acinetobacter baumannii* [36]. Nevertheless, the detection of resistant strains of common bacteria like *E. coli, K. pneumoniae, P. multocida,* and *E. cloacae* highlights the future risk of reduced efficacy for these last-resort options, which must be strictly preserved and used only in extreme cases, guided by susceptibility testing.

The use of empirical antibiotic therapy remains common in clinical practice, as highlighted by our findings. Veterinarians frequently feel obliged to prescribe antibiotics, including for preventive purposes, as a means of protecting themselves both personally and professionally, and to meet owners’ expectations. In fact, animal owners often request antibiotic treatment even when it is not clinically indicated, thereby exerting pressure on the veterinarian, showing how the importance of these drugs is still not fully understood.

**Figure 7:**
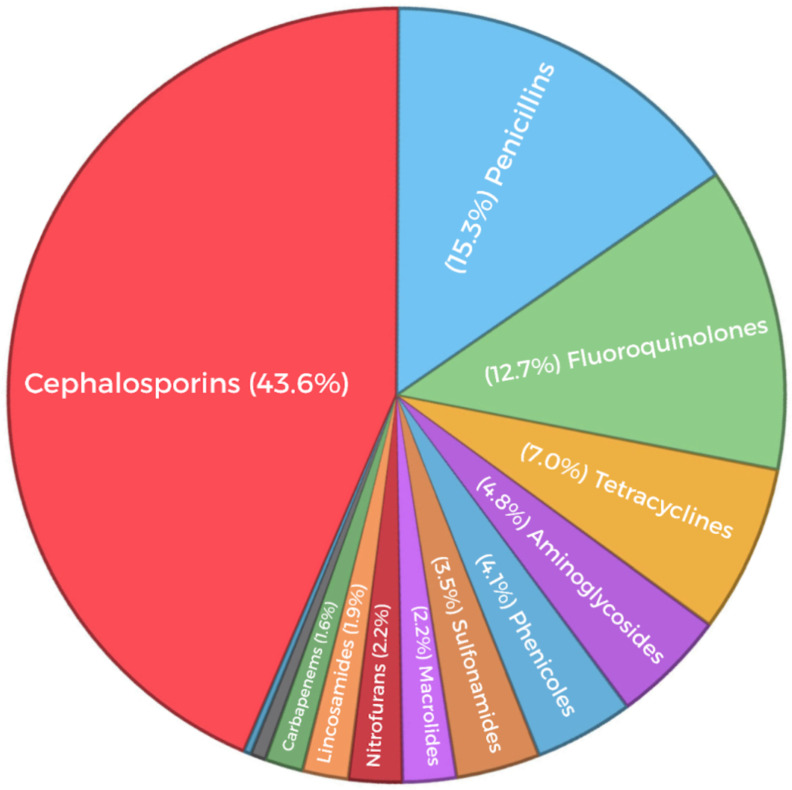
Distribution of antimicrobial resistance in canine isolates (2013–2024), categorized by antimicrobial class. The pie chart illustrates the proportion of resistant isolates attributed to each drug class. Major contributors are cephalosporins (43.6%), penicillins (15.3%), and fluoroquinolones (12.7%). Minor contributors include tetracyclines (7.0%), aminoglycosides (4.8%), phenicols (4.1%), sulfonamides (3.5%), macrolides (2.2%), nitrofurans (2.2%), lincosamides (1.9%) and Carbapenems (1.6%). Values reflect the percentage of resistance events relative to all resistant isolates recorded during the 12-year study period (2013–2024).

Over the 12-year study period, the antibiotic classes with the highest levels of resistance were, in descending order: cephalosporins, penicillins, and fluoroquinolones, as summarized in Figure 7, followed by tetracyclines, aminoglycosides, phenicols, sulfonamides, macrolides, nitrofurans, lincosamides, and carbapenems. These findings are consistent with surveillance and clinic-based studies reporting high resistance proportions to third-generation cephalosporins (often represented in veterinary practice by cefovecin), penicillin, and fluoroquinolones in isolates from dogs and cats [37,38,39].

Several large retrospective and surveillance reports show variable but substantial resistance to cefovecin/third-generation cephalosporins (reported ranges ~6–25% depending on country, year, and sample source) and enrofloxacin/marbofloxacin (reported ranges often 10–30% in clinical *E. coli* and other *Enterobacterales*), which aligns with the predominance of cephalosporin and fluoroquinolone resistance observed in our dataset [37]. Since companion animals are treated with the same antibiotic molecules administered to humans, it is particularly concerning that first-line antibiotics have frequently proven ineffective in the treatment of infections caused by resistant bacteria. In addition, fluoroquinolones, which are third-line antibiotics (AMEG B), are able to exert greater selective pressure on bacterial populations, thereby promoting the development and subsequent spread of antimicrobial resistance, and have also often been found to be ineffective in both veterinary and human medicine [3].

Other studies specifically tracking third-generation cephalosporin-resistant *E. coli* in companion animals report the emergence and regional spread of extended-spectrum β-lactamase (ESBL) producers in both dogs and cats, helping explain sustained high cephalosporin resistance over multi-year periods [38,40].

Comparative analyses indicate some species- and sample-type differences (for example, higher resistance proportions in urinary and skin/ear isolates and occasional higher multidrug-resistance rates in cats versus dogs reported in some cohorts), emphasizing that local clinical ecologies and prescribing practices strongly influence which classes rank highest in resistance [41]. Taken together, our ranking is congruent with the contemporary veterinary AMR literature and underscores the need for continued antimicrobial stewardship, targeted culture, susceptibility testing, and regional surveillance to detect shifts (for example, increases in carbapenem resistance or further expansion of ESBL-producers) that would have major clinical and One Health implications [15,16,29].

The predominance of *E. coli* and *K. pneumoniae* aligns with European [16] and North American [42] data, indicating a gradual shift from staphylococcal dominance to opportunistic Gram-negative organisms such as *Enterobacterales*. Menezes and colleagues [43] identified *K. pneumoniae* as the cause of SSIs dehiscence following sterilization surgery in a dog. This bacterium was resistant to several antibiotics, including third-generation cephalosporins (e.g., cefovecin), fluoroquinolones (enrofloxacin, marbofloxacin, and pradofloxacin), and imipenem. Subsequent tests revealed the presence of carbapenem-resistant *E. coli* in the nasal and rectal cavities. All isolated strains presented similar characteristics of multidrug resistance [44]. Another study carried out by Williams and colleagues demonstrated that surgical site infections occurred in 7% of gastrointestinal surgeries performed on dogs and cats at the University of Pennsylvania Hospital and that the most commonly isolated bacterium was *E. coli*, which was resistant to antibiotics commonly used as first-line treatment in the postoperative period (cefoxitin and cefazolin), following by *Enterobacter cloacae* [45]. A worrying finding comes from the study by Corsini et al., which analyzed the bacteria that predominantly colonize the surface of surgical wounds in canine and feline patients. In fact, 55.26% (21 out of 38 patients) predominantly presented MDR *Staphylococcus* spp. in approximately 86% of cases, and it is essential to emphasize that a high percentage of these bacteria were isolated from the surgeon’s hands (46.15%) and the environment (56.67%). These data suggest the colonizing potential of these bacteria, which are responsible for serious infections in humans [16].

With regard to bite wounds, Kalnins and colleagues analyzed dog bite lesions in 1526 patients from three different hospitals in Australia over a period of 20 years. In 16 cases, microbial cultures were performed, and *E. coli*, *Enterobacter cloacae*, *Pasteurella multocida*, *Proteus mirabilis*, and *Staphylococcus pseudintermedius* were identified in 6.2% of the cases examined [28].

The high rate of ESBL-producing *E. coli* supports previously reported values and reflects the emergence of MDR clones in companion animals with potential zoonotic transmission, emphasizing that AMR is not a species-limited issue but a true One Health concern [29]. Cases of ESBL positivity (4/18 *E. coli* and 7/8 *K. pneumoniae*), MRSA, and MRSP (7/9 *S. aureus* and 3/7 *S. intermedius/pseudintermedius*) further underline the seriousness of the problem. These pathogens not only complicate treatment but also pose zoonotic risks, particularly in cases of close contact, such as wound management or postoperative care at home.

Based on these findings, it is mandatory to hypothesize a series of interventions aimed at planning and promoting an effective ASP strategy. At first, the promotion of routine bacterial cultures must be implemented, and susceptibility testing must guide treatment, not only in chronic or recurrent infections but also in first presentation wounds. According to this, the entire medical and nursing staff must be properly trained to prefer local antibiotic drug administration when possible and avoid systemic administration, and if the treatment is necessary, to choose the molecules according to the most recent AMEG and WHO guidelines. The final aim is to treat the patient, avoiding life-threatening complications but preserving, at the same time, the efficacy of the molecules. Each small animal clinical hospital should write down a specific ASP workflow because, as it was already explained above, the susceptibility testing may be different in their results according to the specific conditions of patients, the previously administered treatments, and the specific epidemiology of the geographical area that must be encountered. Lastly, clinical records have to be complete, and each antibiotic treatment or each variation has to be detailed.

Study limitations are several and must be acknowledged. The retrospective, single-center nature of the study introduced data retrieval challenges and selection bias, limiting generalizability and interpretation. The statistical power was insufficient for multivariate analyses or stratification of AMR-related risk factors due to the small sample size (*n* = 29 culture-positive cases) and the retrospective design, which hindered access to all clinical variables. Due to the lack of a standardized clinical follow-up, it was not possible to establish a correlation between resistance patterns and clinical progression. In addition, variability among veterinarians in terms of experience and years of practice introduced further differences in the therapeutic approach. During the study period, advances in diagnostic technologies and changes in drug legislation led to modifications in the antimicrobial panels tested, which limited direct comparison of AMR trends over time. This study included only hospitalized patients who underwent multiple examinations. Animals with SSIs or bite wounds that did not require hospitalization or repeated examinations rarely had a complete clinical history or sufficient records to be included. This is one of the reasons for the limited number of cases recruited over the study period. The absence of molecular/genetic characterization of isolates (e.g., PCR for mecA/mecC, ESBL, or carbapenemase genes) prevented identification of clonal clusters or potential nosocomial transmission among the patients.

Nonetheless, the decade-long timespan and the clinical relevance of the isolated species confer valuable exploratory significance to this study and lay the groundwork for future research.

Future perspectives include prospective multicenter surveillance projects with larger sample sizes, standardized antimicrobial panels, genomic typing, and integration of clinical variables (e.g., wound severity, administered treatments, and treatment outcomes). This would enable a more comprehensive understanding of AMR dynamics in surgical and bite wounds in companion animals and help trace the dissemination of ESBL and/or MDR clones across regions, not just within a single facility.

Evaluating the clinical and economic impact, as well as associated risk factors, of MDR infections would compare these aspects with the North American context, where Allegretti et al. (2025) [15] described similarities between the risk factors and consequences of MDR infections in both human and veterinary medicine. Finally, integrating veterinary and human AMR data could aid in exploring the bidirectional transmission of resistant bacteria between pets and owners, thus supporting a solid One Health approach to AMR.

## 5. Conclusions

In a referral center in Northwestern Italy, SSIs and bite wounds in dogs and cats analyzed retrospectively over 12 years were dominated by MDR Enterobacterales, with significant rates of ESBL, MRSA, and MRSP and widespread resistance to first-line β-lactams. The clinical outcomes of canine and feline patients infected with this type of bacteria are difficult and expose owners and the shared environment to the spread of bacteria and resistance genes. Aminoglycosides and carbapenems remain effective, but it is essential to remember that the use of these molecules is reserved for human medicine and that their surveillance in veterinary medicine should be understood as an indicator of the possible spread and sharing of AMR/MDR in a zoonotic panorama. This study provides a starting point for assessing bacterial prevalence in a specific Italian geographical area, but similar bacterial analyses should be conducted at the regional, national, and international levels. Continuous molecular surveillance and One Health strategies are essential to contain the evolution of AMR and its spread between species.

## Figures and Tables

**Table 1:** The table reports the absolute number of cases classified by reproductive status in dogs and cats affected by surgical site infections (SSIs) and bite wounds (BWs). Data are presented for each species–condition group (dogs–SSIs, cats–SSIs, dogs–BWs, and cats–BWs).

Group	Female Entire	Female Spayed	Male Entire	Male Neutered
Dogs–SSIs	3	2	8	1
Cats–SSIs	5	0	2	1
Dogs–BWs	0	1	3	0
Cats–BWs	1	0	0	3

**Table 2:** Summary of antimicrobial susceptibility profiles for the three most common bacterial isolates recovered from canine samples (total isolates: *n* = 23; *Escherichia coli* 5/23, *Klebsiella pneumoniae* 4/23, *Enterobacter cloacae* 3/23). Rows list the antimicrobial agents, while columns report the percentage of isolates classified as susceptible (S), intermediate (I), or resistant (R). A dash (–) denotes that 0% of isolates were classified in that category. Interpretive categories are based on MIC-derived breakpoints. The susceptibility testing was interpreted according to CLSI guidelines specific to companion animals and EUCAST guidelines for reference data not available in veterinary medicine.

Drug	*Escherichia coli* (5/23)			*Klebsiella pneumoniae* (4/23)			*Enterobacter cloacae* (3/23)		
	S (%)	I (%)	R (%)	S (%)	I (%)	R (%)	S (%)	I (%)	R (%)
Amikacin	100 (5/5)	–	–	100 (4/4)	–	–	100 (3/3)	–	–
Amoxicillin/clavulanic acid	40 (2/5)	–	60 (3/5)	25 (1/4)	25 (1/4)	50 (2/4)	100 (3/3)	–	–
Ampicillin	20 (1/5)	–	80 (4/5)	–	–	100 (4/4)	–	–	–
Benzylpenicillin	–	–	–	–	–	–	–	–	–
Cefotaxime	–	–	–	–	–	–	–	–	–
Cefovecin	40 (2/5)	–	60 (3/5)	–	25 (1/4)	75 (3/4)	–	33.3 (1/3)	66.6 (2/3)
Cefpodoxime	40 (2/5)	–	60 (3/5)	–	25 (1/4)	75 (3/4)	33.3 (1/3)	–	66.6 (2/3)
Ceftiofur	40 (2/5)	–	60 (3/5)	–	25 (1/4)	75 (3/4)	–	–	100 (3/3)
Ceftriaxone	–	–	–	–	–	–	–	–	–
Cephalexin	40 (2/5)	–	60 (3/5)	–	–	100 (4/4)	–	–	100 (3/3)
Cephalotin	–	–	80 (4/5)	–	–	50 (2/4)	–	–	100 (3/3)
Chloramphenicol	20 (1/5)	–	80 (4/5)	50 (2/4)	–	50 (2/4)	33.3 (1/3)	33.3 (1/3)	33.3 (1/3)
Clindamycin	–	–	–	–	–	–	–	–	–
Doxycycline	20 (1/5)	–	60 (3/5)	25 (1/4)	–	25 (1/4)	66.6 (2/3)	33.3 (1/3)	–
Enrofloxacin	20 (1/5)	–	80 (4/5)	–	25 (1/4)	75 (3/4)	33.3 (1/3)	33.3 (1/3)	33.3 (1/3)
Erythromycin	–	–	–	–	–	–	–	–	–
ESBL	2 neg	–	3 pos	1 neg	–	3 pos	not evaluated		
Gentamicin	80 (4/5)	–	20 (1/5)	75 (3/4)	–	25 (1/4)	100 (3/3)	–	–
Imipenem	100 (5/5)	–	–	100 (4/4)	–	–	66.6 (2/3)	–	33.3 (1/3)
Kanamycin	–	–	–	–	–	–	–	–	–
Marbofloxacin	20 (1/5)	–	80 (4/5)	25 (1/4)	–	75 (3/4)	33.3 (1/3)	–	66.6 (2/3)
Neomycin	80 (4/5)	–	–	25 (1/4)	25 (1/4)	–	100 (3/3)	–	–
Nitrofurantoin	100 (5/5)	–	–	25 (1/4)	75 (3/4)	–	33.3 (1/3)	33.3 (1/3)	33.3 (1/3)
Oxacillin	–	–	–	–	–	–	–	–	–
Pradofloxacin	20 (1/5)	–	80 (4/5)	–	50 (2/4)	50 (2/4)	33.3 (1/3)	33.3 (1/3)	33.3 (1/3)
Tetracycline	40 (2/5)	–	60 (3/5)	25 (1/4)	–	75 (3/4)	100 (3/3)	–	–
Trimethoprim/sulfamethoxazole	40 (2/5)	–	60 (3/5)	50 (2/4)	–	50 (2/4)	66.6 (2/3)	–	33.3 (1/3)

**Table 3:** Summary of antimicrobial susceptibility profiles for the main bacterial isolates recovered from feline samples (total isolates: *n* = 12; *Escherichia coli* 2/12, *Pasteurella multocida* 2/12, *Enterobacter cloacae* 2/12). Rows list the antimicrobial agents, while columns report the percentage of isolates classified as susceptible (S), intermediate (I), or resistant (R). A dash (–) denotes that 0% of isolates were classified in that category. Interpretive categories are based on MIC-derived breakpoints. The susceptibility testing was interpreted according to CLSI guidelines specific to companion animals and EUCAST guidelines for reference data not available in veterinary medicine.

Drug	*Escherichia coli* (2/12)			*Pasteurella multocida* (2/12)			*Enterobacter cloacae* (2/12)		
	S (%)	I (%)	R (%)	S (%)	I (%)	R (%)	S (%)	I (%)	R (%)
Amikacin	100 (2/2)	–	–	–	–	–	100 (2/2)	–	–
Amoxicillin/clavulanic acid	50 (1/2)	–	50 (1/2)	100 (2/2)	–	–	–	–	100 (2/2)
Ampicillin	–	–	100 (2/2)	50 (1/2)	–	–	–	–	–
Benzylpenicillin	–	–	–	–	–	–	–	–	–
Cefotaxime	–	–	–	–	–	–	–	–	–
Cefovecin	50 (1/2)	–	50 (1/2)	50 (1/2)	–	–	50 (1/2)	–	50 (1/2)
Cefpodoxime	50 (1/2)	–	50 (1/2)	50 (1/2)	–	–	–	–	100 (2/2)
Ceftiofur	–	50 (1/2)	50 (1/2)	100 (2/2)	–	–	–	–	100 (2/2)
Ceftriaxone	–	–	–	–	–	–	–	–	–
Cephalexin	–	–	100 (2/2)	100 (2/2)	–	–	–	–	100 (2/2)
Cephalotin	–	50 (1/2)	50 (1/2)	–	–	–	–	–	–
Chloramphenicol	50 (1/2)	–	50 (1/2)	100 (2/2)	–	–	–	–	100 (2/2)
Clindamycin	–	–	–	–	–	–	–	–	–
Doxycycline	100 (2/2)	–	–	50 (1/2)	–	–	–	–	–
Enrofloxacin	50 (1/2)	–	50 (1/2)	100 (2/2)	–	–	–	–	100 (2/2)
Erythromycin	–	–	–	–	–	–	–	–	–
ESBL	1 neg	–	1 pos		not evaluated		not evaluated		
Gentamicin	100 (2/2)	–	–	50 (1/2)	50 (1/2)	–	–	–	100 (2/2)
Imipenem	50 (1/2)	–	–	50 (1/2)	–	–	50 (1/2)	–	50 (1/2)
Kanamycin	–	–	–	–	–	–	–	–	–
Marbofloxacin	50 (1/2)	–	50 (1/2)	100 (2/2)	–	–	–	–	100 (2/2)
Neomycin	100 (2/2)	–	–	100 (2/2)	–	–	–	–	–
Nitrofurantoin	100 (2/2)	–	–	–	–	–	–	–	100 (2/2)
Oxacillin	–	–	–	–	–	–	–	–	–
Pradofloxacin	50 (1/2)	–	50 (1/2)	100 (2/2)	–	–	50 (1/2)	–	50 (1/2)
Tetracycline	100 (2/2)	–	–	100 (2/2)	–	–	–	–	100 (2/2)
Trimethoprim/sulfamethoxazole	50 (1/2)	–	50 (1/2)	100 (2/2)	–	–	50 (1/2)	–	50 (1/2)

**Table 4:** Summary of the antibiotic treatments, divided by species, surgical site infections (SSIs), and bite wounds (BWs). IV = intravenous route of administration; PO = *per os* route of administration.

**SSIs**	**Dogs (*n* = 14)**		**Cats (*n* = 7)**	
	**Drug—Administration route**	**N° of treatments**	**Drug—Administration route**	**N° of treatments**
	Ampicillin—IV	8	Ampicillin—IV	5
	Marbofloxacin—PO	2	Marbofloxacin—PO	2
	Amoxicillin + clavulanic acid—PO	4	-	-
**BWs**	**Dogs (*n* = 4)**		**Cats (*n* = 4)**	
	**Drug—Administration route**	**N° of treatments**	**Drug—Administration route**	**N° of treatments**
	Marbofloxacin—PO	4	Cefovecin—PO	4

## Data Availability

Data are unavailable due to the privacy of the patients and owners involved.
